# Supplemental *Clostridium butyricum* modulates lipid metabolism by reshaping the gut microbiota composition and bile acid profile in IUGR suckling piglets

**DOI:** 10.1186/s40104-023-00828-1

**Published:** 2023-03-13

**Authors:** Xin Zhang, Yang Yun, Zheng Lai, Shuli Ji, Ge Yu, Zechen Xie, Hao Zhang, Xiang Zhong, Tian Wang, Lili Zhang

**Affiliations:** College of Animal Science and Technology, Nanjing Agricultural University, 210095 Nanjing, Jiangsu China

**Keywords:** Bile acid, *Clostridium butyricum*, Gut microbiota, Intrauterine growth restriction, Lipid metabolism, Suckling piglet

## Abstract

**Background:**

Intrauterine growth restriction (IUGR) can cause lipid disorders in infants and have long-term adverse effects on their growth and development. *Clostridium butyricum* (*C. butyricum*), a kind of emerging probiotics, has been reported to effectively attenuate lipid metabolism dysfunctions. Therefore, the objective of this study was to investigate the effects of *C. butyricum* supplementation on hepatic lipid disorders in IUGR suckling piglets.

**Methods:**

Sixteen IUGR and eight normal birth weight (NBW) neonatal male piglets were used in this study. From d 3 to d 24, in addition to drinking milk, the eight NBW piglets (NBW-CON group, *n* = 8) and eight IUGR piglets (IUGR-CON group, *n* = 8) were given 10 mL sterile saline once a day, while the remaining IUGR piglets (IUGR-CB group, *n* = 8) were orally administered *C. butyricum* at a dose of 2 × 10^8^ colony-forming units (CFU)/kg body weight (suspended in 10 mL sterile saline) at the same frequency.

**Results:**

The IUGR-CON piglets exhibited restricted growth, impaired hepatic morphology, disordered lipid metabolism, increased abundance of opportunistic pathogens and altered ileum and liver bile acid (BA) profiles. However, *C. butyricum* supplementation reshaped the gut microbiota of the IUGR-CB piglets, characterized by a decreased abundance of opportunistic pathogens in the ileum, including *Streptococcus* and *Enterococcus*. The decrease in these bile salt hydrolase (BSH)-producing microbes increased the content of conjugated BAs, which could be transported to the liver and function as signaling molecules to activate liver X receptor α (LXRα) and farnesoid X receptor (FXR). This activation effectively accelerated the synthesis and oxidation of fatty acids and down-regulated the total cholesterol level by decreasing the synthesis and promoting the efflux of cholesterol. As a result, the growth performance and morphological structure of the liver improved in the IUGR piglets.

**Conclusion:**

These results indicate that *C. butyricum* supplementation in IUGR suckling piglets could decrease the abundance of BSH-producing microbes (*Streptococcus* and *Enterococcus*). This decrease altered the ileum and liver BA profiles and consequently activated the expression of hepatic LXRα and FXR. The activation of these two signaling molecules could effectively normalize the lipid metabolism and improve the growth performance of IUGR suckling piglets.

**Supplementary Information:**

The online version contains supplementary material available at 10.1186/s40104-023-00828-1.

## Background

From the moment of birth, a newborn must begin adapting to a different nutritional environment and obtaining energy from milk [1]. From d 3 to weaning, fat predominates in porcine milk. Due to its high energy value, this fat provides approximately 60% of the energy required for the growth of newborn piglets [2, 3]. Thus, a functional and efficient lipid metabolic system is crucial for the growth and development of newborns during the suckling period. However, piglets with intrauterine growth restriction (IUGR) show abnormal lipid metabolism and impaired growth performance, which severely impacts their health and results in considerable losses in animal production [4, 5]. Therefore, it is necessary to identify a method to regulate lipid metabolism in IUGR suckling piglets.

Emerging as a kind of probiotic, *C. butyricum* is a Gram-positive anaerobe that produces butyric acid. It is one of the earliest microbial colonizers in infants and primarily exists in the distal small intestine and colon of animals [6–8]. *C. butyricum* also exhibits resistance to acidic pH levels, high temperature and bile salts. Therefore, *C. butyricum* is regarded as a useful and safe additive [9], and previous studies have shown that it can improve growth performance, protect against pathogenic bacteria and strengthen immunity in weaned piglets [10–12].

Additionally, *C. butyricum* plays a role in the regulation of lipid metabolism, and this feature has been demonstrated in models of aged laying hens, high fat diet (HFD) mice and corticosterone-challenged ducks [13–15]. These previous studies showed that *C. butyricum* could regulate fatty acid (FA) metabolism by modifying the expression of lipogenesis-related genes, such as acetyl-CoA carboxylase (ACC), and lipolysis-related genes, such as peroxisome proliferator activated receptor alpha (PPARα) [14, 16]. *C. butyricum* has also been shown to modulate cholesterol metabolism by elevating the mRNA expression of *CYP7A1* and *CYP8B1* to increase cholesterol efflux [15]. However, it remains unclear whether *C. butyricum* supplementation could relieve the disordered lipid metabolism of IUGR suckling piglets.

The gut microbiota, as a crucial regulator of host metabolism, has the capacity to produce or modulate metabolites that function as metabolic substrates and signaling molecules in the host [17]. Disruptions to the gut microbiota may lead to various metabolic disorders including obesity, type 2 diabetes and malnutrition [18]. Previous studies revealed that IUGR can disturb the micro-ecological equilibrium of the gut and, as a result, negatively impact normal metabolic pathways [19, 20].

An increasing body of evidence indicates that the metabolic regulation of the gut microbiota is realized through the gut-liver axis [21], and as an important metabolite of the gut microbiota, bile acid (BA) can function as a signaling molecule and exert an impact on host metabolism [22]. During BA metabolism, the bile acid-activated receptors farnesoid X receptor (FXR) and liver X receptor (LXR) are highly expressed in the enterohepatic tissues, and both of these intracellular sensors can be activated to maintain lipid homeostasis through the gut-liver axis [23, 24]. In addition, others have shown that *C. butyricum* treatment can alter the BA profile of the liver and ileum and simultaneously affect the intestinal microbiota composition of the host [14, 15]. These substantial findings suggest that the addition of *C. butyricum* may exert an effect on BA metabolism via the gut microbiota. Nevertheless, how *C. butyricum* supplementation influences lipid metabolism and the gut microbiota–BA metabolism relationship still requires further exploration.

Therefore, in the present study, the aims were to determine whether *C. butyricum* supplementation could be an effective means of regulating lipid metabolism in IUGR piglets during the suckling period and to explore the underlying mechanism from the perspective of the gut-liver axis.

## Materials and methods

### Animals and experimental design

All experiments were conducted in accordance with the guidelines of the Institutional Animal Care and Use Committee of Nanjing Agricultural University. Forty healthy sows (Landrace × Yorkshire) in their third parity and with similar expected dates of confinement (≤ 3 d) were initially selected. After screening, eight sows that had similar litter sizes (12.13 ± 0.60) and met the selection criteria for IUGR were selected. The newborn piglets (Duroc × [Landrace × Yorkshire]) that weighed within 0.5 standard deviation (SD) of the mean birth weight (BW) of the littermates were defined as normal birth weight (NBW), whereas those with 2 SD lower BW were defined as IUGR [25, 26]. According to this criterion, two IUGR (0.90 ± 0.08 kg) and one NBW (1.62 ± 0.10 kg) male piglets were chosen from each sow. The sixteen IUGR and eight NBW piglets were then randomly allocated to three groups: the NBW-CON group (NBW piglets that received 10 mL sterile saline per day, *n* = 8), the IUGR-CON group (IUGR piglets that received 10 mL sterile saline per day, *n* = 8) and the IUGR-CB group (IUGR piglets that received 10 mL bacterial fluid per day, *n* = 8). After colostrum feeding, all piglets were randomly assigned to four sows (6 piglets/sow; NBW-CON = 2, IUGR-CON = 2, IUGR-CB = 2) with similar physiological condition for lactation. When 2 d of adaptation finished, the gavage trial was conducted from d 3 to d 24. The dose of *C. butyricum* in bacterial fluid was 2 × 10^8^ CFU/kg BW, and the BW of the piglets was measured every 3 d. All piglets were kept in lactation crates and nursed by sows, and sow milk was the only available dietary sustenance for the piglets during the study.

The *C. butyricum* used in the study was provided by Qingdao Vland Biological Technology Co., Ltd. (Qingdao, Shandong, China). The spore count was 5 × 10^9^ CFU/g. The strain was *C. butyricum* wl-53, which was initially isolated from the feces of healthy chickens and was conserved in the China Center for Type Culture Collection (CCTCC No. M2019252, Wuhan, Hubei, China).

### Sample collection

Early in the morning of d 24, the piglets were weighed and the measurements were recorded as the final body weight (FBW) before blood collection. Then, blood sample was collected from the precaval vein of each piglet before sacrifice. Plasma was obtained by centrifugation at 3000 × *g* for 15 min at 4 °C, and stored at −80 °C for subsequent analysis. All piglets were killed by exsanguination after electrical stunning, after which fresh samples of liver and ileum chyme were immediately collected. After flushing the liver with saline, liver samples about 1 cm^3^ in size were collected from the left lobe and fixed in 4% paraformaldehyde solution for histological analysis. The remaining parts of the liver and the samples of chyme collected from the ileum were snap-frozen in liquid nitrogen and then stored at −80 °C for further analysis.

### Histopathology

After being fixed in 4% paraformaldehyde for 24 h, the liver samples were dehydrated using an ethanol concentration gradient and then embedded in paraffin. These paraffin blocks were sliced into 5 μm sections, and the sections were stained with hematoxylin and eosin. The hepatic morphology was observed using a light microscopy (Nikon 80i, Tokyo, Japan).

### Biochemical assay of serum samples

Commercial assay kits were used to determine the triglyceride (TG; #A110-1-1), nonesterified free fatty acids (NEFA; #A042-1-1), total cholesterol (TC; #A111-1-1), total bile acid (TBA; #E003-2-1), high-density lipoprotein cholesterol (HDL-C; #A112-1-1), low-density lipoprotein cholesterol (LDL-C; #A113-1-1) and glucose (GLU; #A154-1-1) content, according to the manufacturer’s instructions (Nanjing JianCheng Bioengineering Institute, Nanjing, Jiangsu, China).

### Determination of hepatic metabolite concentration

Commercial assay kits were used to determine the TG (#A110-1-1), lipoprotein lipase (LPL; #A067-1-2), hepatic lipase (HL; #A067-1-2), TC (#A111-1-1), TBA (#E003-2-1) and total protein (TP; #A045-4-2) content, according to the manufacturer’s instructions (Nanjing Jiancheng Bioengineering Institute).

The 3-hydroxy-3-methylglutaryl-CoA reductase (HMGCR; #RX500243P) and very low-density lipoprotein (VLDL; #RX500284P) levels were detected using enzyme-linked immunoassay (ELISA) kits for swine from Quanzhou Ruixin Biological Technology Co., Ltd. (Quanzhou, Fujian, China), following the manufacturer’s instructions.

### RNA isolation and quantitative real-time polymerase chain reaction (PCR) analysis

Total RNA was extracted from the ileum (mucosal) and liver samples using the Total RNA Extraction Reagent (Vazyme Biotechnology, Nanjing, Jiangsu, China) and quantified using an ND-2000 micro spectrophotometer (Thermo Fisher Scientific, Waltham, MA, USA). After the determination of RNA quality and concentration, 1 µg of total RNA was reverse-transcribed into complementary DNA (cDNA) using the HiScript III RT SuperMix Reagent (Vazyme Biotechnology), following the manufacturer’s instructions. The mRNA expression levels of specific genes were quantified via real-time polymerase chain reaction (PCR) using the SYBR qPCR Master Mix (Vazyme Biotechnology) and the QuantStudio 5 Real-Time PCR System (Thermo Scientific, Wilmington, DE, USA). The SYBR Green PCR reaction mixture consisted of 10 µL TB Green Premix Ex Taq, 0.4 µL ROX Reference Dye II, 2 µL cDNA template, 0.4 µL of each primer (total 0.8 µL, 10 µmol/L) and 6.8 µL of double-distilled H_2_O. The reaction conditions were as follows: pre-run at 95 °C for 30 s, 40 denaturation cycles at 95 °C for 10 s and annealing at 60 °C for 30 s. Each sample was run in triplicate. The relative mRNA expression levels were analyzed via the 2^−ΔΔCt^ method after normalization against *β-actin*, and the results displayed a similar trend when *GAPDH* served as the housekeeping gene.

### Protein extraction and western blot assay

TP was isolated from the frozen liver samples using a lysis buffer containing protease inhibitors (Beyotime Institute of Biotechnology, Nantong, Jiangsu, China). The protein concentration was measured using a BCA Protein Assay Kit (Beyotime Institute of Biotechnology). Equal amounts of TP (20 µg) were subjected to electrophoresis in 4%–20% SDS-PAGE and then transferred to PVDF membranes activated by methanol. After blocking with 5% fat-free dry milk in TBST (0.05% Tween-20, 100 mmol/L Tris–HCl, and 150 mmol/L NaCl, pH 7.5) at room temperature for 2 h, the membranes were incubated overnight at 4 °C with primary antibodies that target specific proteins, including β-actin (#20536-1-AP; Proteintech, Chicago, IL, USA), NR1H4 (#25055-1-AP; Proteintech), NR1H3 (#14351-1-AP; Proteintech), PPARα (#66826-1-Ig; Proteintech) and CYP7A1(#AF6657; Beyotime Institute of Biotechnology) and CYP27A1(#14739-1-AP; Proteintech). The blots were washed in TBST three times and incubated for 1.5 h at room temperature with a secondary antibody: alkaline phosphatase-conjugated goat anti-rabbit IgG or anti-mouse IgG (#BL023A and #BL021A; Biosharp, Hefei, Anhui, China). Finally, the blots were washed with TBST three times before protein detection using an enhanced chemiluminescence reagent (#BL520A; Biosharp) and visualisation on a ChemiDocTM Imaging System (BIO-RAD, Hercules, CA, USA). Protein band intensity was quantified using ImageJ 1.42 q software (NIH, Bethesda, MD, USA).

### 16S rRNA analysis of the ileal microbial community

Total bacterial DNA was extracted from the ileum chyme samples using the TIANamp Stool DNA Kit (Tiangen Biotech, Beijing, China), according to the manufacturer’s guidance. The V3–V4 region of the bacterial 16S rRNA genes was amplified using the specific primers 341 F/806R (341 F: 5’-ACTCCTACGGGAGGCAGCAG-3’; 806R: 5’-GGACTACHVGGGTWTCTAAT-3’). The PCR reaction involved a thermal cycle consisting of initial denaturation at 94 °C for 3 min, then 30 cycles of 94 ℃ for 30 s, 56 °C for 45 s, and 72 °C for 45 s, and a final extension step for 10 min at 72 °C. PCR enrichment was performed in a 50-µL reaction containing 30 ng template, fusion PCR primer and PCR master mix. The PCR products were purified with AmpureXP beads and eluted in an elution buffer. Libraries were qualified using the Agilent 2100 Bioanalyzer (Agilent, Santa Clara, CA, USA). The validated libraries were sequenced using the Illumina MiSeq platform (BGI, Shenzhen, Guangdong, China), following the standard pipelines of Illumina and generating 2 × 300 bp paired-end reads.

Raw reads were filtered to remove adaptors and low-quality ambiguous bases, and then paired-end reads were added to tags by the Fast Length Adjustment of Short reads program (V 1.2.11) to generate the tags. Subsequently, the clean tags were assigned to operational taxonomic units with a threshold of 97% identity by UPARSE (V 7.0.1090), and the chimeric sequences were identified and eliminated using UCHIME (V 4.2.40). Species annotation analysis was performed by an RDP Classifier (V 2.2) based on the Greengene database (V 201,305) with a minimum confidence threshold of 80%. The rarefaction curve, species accumulation curve and Shannon, Simpson and Chao indices were calculated using RStudio software (V 3.5.3) with the vegan package. To estimate beta diversity, principal coordinates analysis (PCoA) based on the Bray-Curtis distance and an analysis of similarities (ANOSIM) were conducted to compare the differences between the treatments using RStudio software (V 3.5.3).

### Targeted metabolome analysis of intestinal and liver bile acids (BAs)

The BA content of the ileal chyme and the liver was determined using ultra-high performance liquid chromatography–mass spectrometry (UPLC/MS, ACQUITY UPLC-Xevo TQ-S, Waters Corp., Milford, MA, USA). In brief, approximately 10 mg of each freeze-dried sample was added to an Eppendorf tube along with 10 µL internal standard, 190 µL acetonitrile/methanol (v/v = 8:2) and 25 mg pre-cooling grinding beads. The sample was homogenized, and the homogenate was centrifuged at 13,500 r/min for 20 min at 4 ℃ (Microfuge 20R, Beckman Coulter, Inc., Indianapolis, IN, USA). Next, 10 µL of the resultant supernatant was removed and diluted with 45 µL acetonitrile/methanol (v/v = 8:2) and ultrapure water. Then, 5 µL of the diluent was applied to the UPLC/MS system for BA quantification. An ACQUITY UPLC Cortecs C18 1.6 μm analytical column (2.1 mm × 100 mm) heated to 30 ℃ was used for chromatographic separation. The gradient system consisted of Solvent A (10 mmol/L ammonium acetate with 0.25% acetate acid) and Solvent B (acetonitrile:methanol:isopropanol = 8:1:1) at a flow rate of 0.4 mL/min. The other parameters were set as follows: capillary voltage = 2.0 kV, ion source temperature = 150 ℃, desolvation temperature = 550 ℃, desolvation flow = 1000 L/h. BA standards were purchased from Steraloids, Co. Ltd (Newport, RI, USA) and TRC Chemicals, Co. Ltd (Toronto, ON, Canada). The mixed reference standards were obtained by dissolving each BA reference standard in methanol.

### Statistical analysis

The data were analyzed using SPSS 25.0 statistical software (ver. 25.0 for Windows, SPSS Inc., Chicago, IL, USA). Statistical differences in serum and hepatic biochemical indices, hepatic gene and protein expression levels and intestinal and hepatic BA contents were determined by one-way ANOVA followed by Tukey’s test when *F* was significant. The Kruskal–Wallis test was used to detect differences in the relative abundance of bacteria among these groups. A *P*-value < 0.05 was considered statistically significant. The results are presented as mean ± SE.

## Results

### Growth performance

The piglets in the IUGR-CON group had lower FBWs than those in the NBW-CON group (*P* < 0.05), indicating that IUGR decreased the FBW. In contrast, in the IUGR-CB group, *C. butyricum* supplementation improved (*P* < 0.05) the growth performance of these piglets, and the average daily gain (ADG) and FBW were increased by 41.60% and 30.57%, respectively (Table 1).

**Table 1:** Effect of supplemental *C. butyricum* on the growth performance of IUGR suckling piglets from 3 to 24 days of age

Items^1^	NBW-CON^2^	IUGR-CON	IUGR-CB	*P*-values		
				1	2	3
IBW, kg	2.24 ± 0.03	1.51 ± 0.11^*^	1.52 ± 0.12^*^	< 0.001	< 0.001	0.998
FBW, kg	7.16 ± 0.19	5.66 ± 0.40^*^	7.39 ± 0.26^#^	0.005	0.847	0.001
ADG, g/d	234.11 ± 8.56	197.32 ± 16.42	279.40 ± 9.77^*^^#^	0.103	0.038	< 0.001

All data are presented as mean ± SE (*n* = 8). Significant difference is depicted as ^*^P < 0.05 when compared with NBW-CON group, ^#^*P *< 0.05 when compared with IUGR-CON group. Contrast: (1) NBW-CON versus IUGR-CON; (2) NBW-CON versus IUGR-CB; (3) IUGR-CON versus IUGR-CB

^1^IBW, initial body weight; FBW, final body weight; ADG, average daily gain

^2^NBW-CON, piglets with normal birth weight; IUGR-CON, piglets with intrauterine growth restriction; IUGR-CB, piglets with intrauterine growth restriction supplemented with *Clostridium butyricum*

### Histopathological analysis

**Fig. 1:**
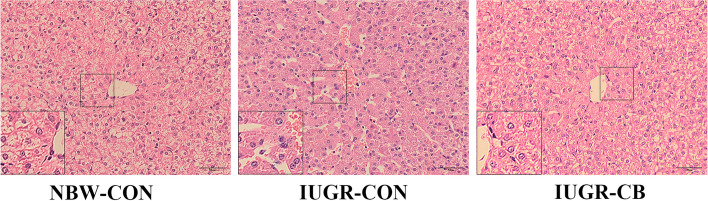
Effect of supplemental *C. butyricum* on hepatic histomorphology of IUGR suckling piglets. All samples were stained with hematoxylin and eosin (Low magnification: ×400, High magnification: ×800, Bars: 50 μm). NBW-CON, piglets with normal birth weight; IUGR-CON, piglets with intrauterine growth restriction; IUGR-CB, piglets with intrauterine growth restriction supplemented with *Clostridium butyricum*

As shown in Fig. 1, liver samples from IUGR-CON group piglets exhibited a congestive central vein, loosely arranged cords, dilated sinusoids and fewer lipid droplets in the hepatic lobule. However, *C. butyricum* supplementation effectively ameliorated these conditions, and normally oriented liver morphology was observed in the liver samples from IUGR-CB group piglets.

### Fatty acid (FA) metabolism

#### Alterations in the FA metabolites content and the corresponding enzymes in the serum and liver

As shown in Table 2, a higher (*P* < 0.05) level of serum NEFA and lower (*P* < 0.05) hepatic HL and TL levels were found in the IUGR-CON group compared to the NBW-CON group, indicating that IUGR induced these conditions. However, in the IUGR-CB group, *C. butyricum* treatment resulted in significantly less (*P* < 0.05) NEFA accumulation in the serum and elevated (*P* < 0.001) hepatic levels of TG and HL.

**Table 2:** Effect of supplemental *C. butyricum* on the content of FA metabolites and their corresponding enzymes in the serum and liver of IUGR suckling piglets

Items^1^	NBW-CON^2^	IUGR-CON	IUGR-CB	*P*-values		
				1	2	3
Serum						
TG, mmol/L	1.27 ± 0.10	1.25 ± 0.18	1.25 ± 0.07	0.993	0.991	1.000
NEFA, µmol/L	1.49 ± 0.14	1.94 ± 0.04^*^	0.95 ± 0.11^*^^#^	0.027	0.005	< 0.001
Liver						
TG, µmol/gprot	89.66 ± 4.53	74.37 ± 5.92	108.83 ± 3.98^*^^#^	0.092	0.029	< 0.001
HL, U/gprot	580.11 ± 14.66	502.69 ± 9.49^*^	621.34 ± 5.99^*^^#^	< 0.001	0.045	< 0.001
LPL, U/gprot	679.73 ± 14.25	675.03 ± 17.36	694.09 ± 20.13	0.980	0.830	0.723
TL, U/gprot	1259.84 ± 19.35	1177.72 ± 11.57^*^	1315.43 ± 24.70^#^	0.018	0.128	< 0.001
VLDL, mmol/gprot	0.57 ± 0.04	0.52 ± 0.02	0.57 ± 0.02	0.537	0.996	0.587

All data are presented as mean ± SE (*n* = 8). Significant difference is depicted as ^*^P < 0.05 when compared with NBW-CON group, ^#^*P* < 0.05 when compared with IUGR-CON group. Contrast: (1) NBW-CON versus IUGR-CON; (2) NBW-CON versus IUGR-CB; (3) IUGR-CON versus IUGR-CB

^1^TG, triglyceride; NEFA, non-esterified fatty acid; HL, hepatic lipase; LPL, lipoprotein lipase; TL, total lipase; VLDL, very low-density lipoprotein

^2^NBW-CON, piglets with normal birth weight; IUGR-CON, piglets with intrauterine growth restriction; IUGR-CB, piglets with intrauterine growth restriction supplemented with *Clostridium butyricum*

#### Gene expression involved in FA uptake, synthesis and oxidation

**Fig. 2:**
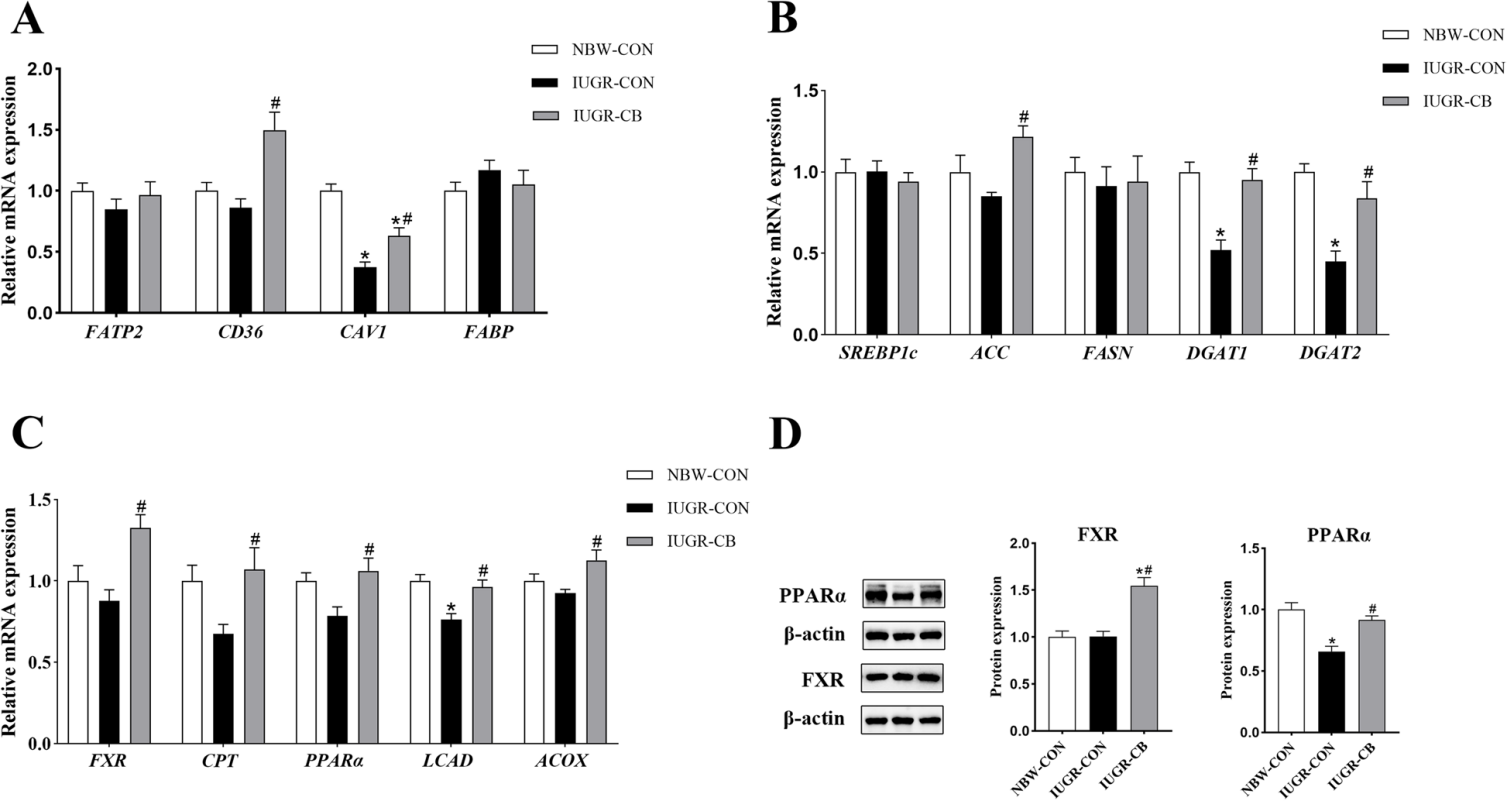
Effect of supplemental *C. butyricum* on FA metabolism of IUGR suckling piglets. **A** The mRNA abundance of genes related to FA uptake and transport. **B** The mRNA abundance of genes related to FA synthesis. **C** The mRNA abundance of genes related to FA oxidation. **D** The protein levels of FXR and PPARα. The column and its bar represented the means value and SE (*n* = 8), respectively. Significant difference is depicted as ^*^P < 0.05 when compared with NBW-CON, ^#^*P* < 0.05 when compared with IUGR-CON group. FATP2, fatty acid transport protein 2; CD36, cluster of differentiation 36; CAV1, caveolin 1; FABP, fatty acid binding protein; SREBP1c, sterol regulatory element-binding protein 1c; ACC, acetyl-CoA carboxylase; FASN, fatty acid synthase; DGAT1, diacylglycerol transferase 1; DGAT2, diacylglycerol transferase 2; FXR, farnesoid X receptor; CPT1, carnitine palmitoyltransferase 1; PPARα, peroxisome proliferator-activated receptor α; LCAD, long-chain acyl-CoA dehydrogenase; ACOX, acyl-CoA oxidase; NBW-CON, piglets with normal birth weight; IUGR-CON, piglets with intrauterine growth restriction; IUGR-CB, piglets with intrauterine growth restriction supplemented with *Clostridium butyricum*

Regarding FA uptake, piglets in the IUGR-CON group had lower (*P* < 0.05) *CAV1* mRNA expression levels than piglets in the NBW-CON group. Piglets in the IUGR-CB group had significantly higher (*P* < 0.05) levels of *CD36* and *CAV1* mRNA expression compared to piglets in the IUGR-CON group (Fig. 2A).

Regarding FA synthesis, IUGR significantly decreased the gene expression of *DGAT1* and *DGAT2* (IUGR-CON group vs. NBW-CON group), and *C. butyricum* supplementation resulted in increased expression of *ACC*, *DGAT1* and *DGAT2* in the IUGR-CB group (Fig. 2B).

In terms of fatty acid oxidation (FAO), *LCAD* mRNA expression was significantly lower in the IUGR-CON group compared to that in the NBW-CON group. In contrast, compared to the IUGR-CON group, expression of *FXR*, *CPT1*, *PPARα*, *LCAD* and *ACOX* were all significantly higher in the IUGR-CB group (*P* < 0.05) (Fig. 2C).

#### Protein expression related to FA transport and metabolism

As shown in Fig. 2D, the expression of PPARα was significantly lower (*P* < 0.05) in the IUGR-CON group compared to that in the NBW-CON group. In contrast, IUGR piglets treated with *C. butyricum* exhibited higher (*P* < 0.05) expression of FXR and PPARα than IUGR piglets that did not receive *C. butyricum*.

### Cholesterol and BA metabolism

#### Alterations in cholesterol and BA metabolites in serum and liver

As shown in Table 3, the IUGR-CON group piglets had significantly lower (*P* < 0.05) serum HDL-C and hepatic TBA concentrations and higher (*P* < 0.05) hepatic TC levels than the NBW-CON group piglets. In contrast, piglets that received *C. butyricum* had elevated (*P* < 0.05) serum HDL-C and hepatic TBA concentrations and lower (*P* < 0.05) hepatic TC levels, compared to the IUGR-CON group piglets.

**Table 3:** Effect of supplemental *C. butyricum* on the content of TC and its metabolite in the serum and liver of IUGR suckling piglets

Items^1^	NBW-CON^2^	IUGR-CON	IUGR-CB	*P-*values		
				1	2	3
Serum						
TC, mmol/L	6.83 ± 0.57	5.28 ± 0.72	6.38 ± 0.57	0.208	0.865	0.443
HDL-C, mmol/L	3.28 ± 0.17	2.47 ± 0.19^*^	3.54 ± 0.23^#^	0.022	0.636	0.003
LDL-C, mmol/L	3.55 ± 0.31	2.80 ± 0.29	2.83 ± 0.23	0.165	0.186	0.997
Liver						
TC, mmol/gprot	56.27 ± 3.62	72.54 ± 2.54^*^	58.11 ± 3.12^#^	0.004	0.910	0.010
TBA, µmol/gprot	7.12 ± 0.48	5.62 ± 0.33^*^	7.21 ± 0.40^#^	0.041	0.988	0.030

All data are presented as mean ± SE (*n* = 8). Significant difference is depicted as ^*^P < 0.05 when compared with NBW-CON, ^#^*P* < 0.05 when compared with IUGR-CON group. Contrast: (1) NBW-CON versus IUGR-CON; (2) NBW-CON versus IUGR-CB; (3) IUGR-CON versus IUGR-CB

^1^TC, total cholesterol; HDL-C, high density lipoprotein cholesterol; LDL-C, low density lipoprotein cholesterol; TBA, total bile acid

^2^NBW-CON, piglets with normal birth weight; IUGR-CON, piglets with intrauterine growth restriction; IUGR-CB, piglets with intrauterine growth restriction supplemented with *Clostridium butyricum*

#### Expression of genes associated with cholesterol and BA metabolism

**Fig. 3:**
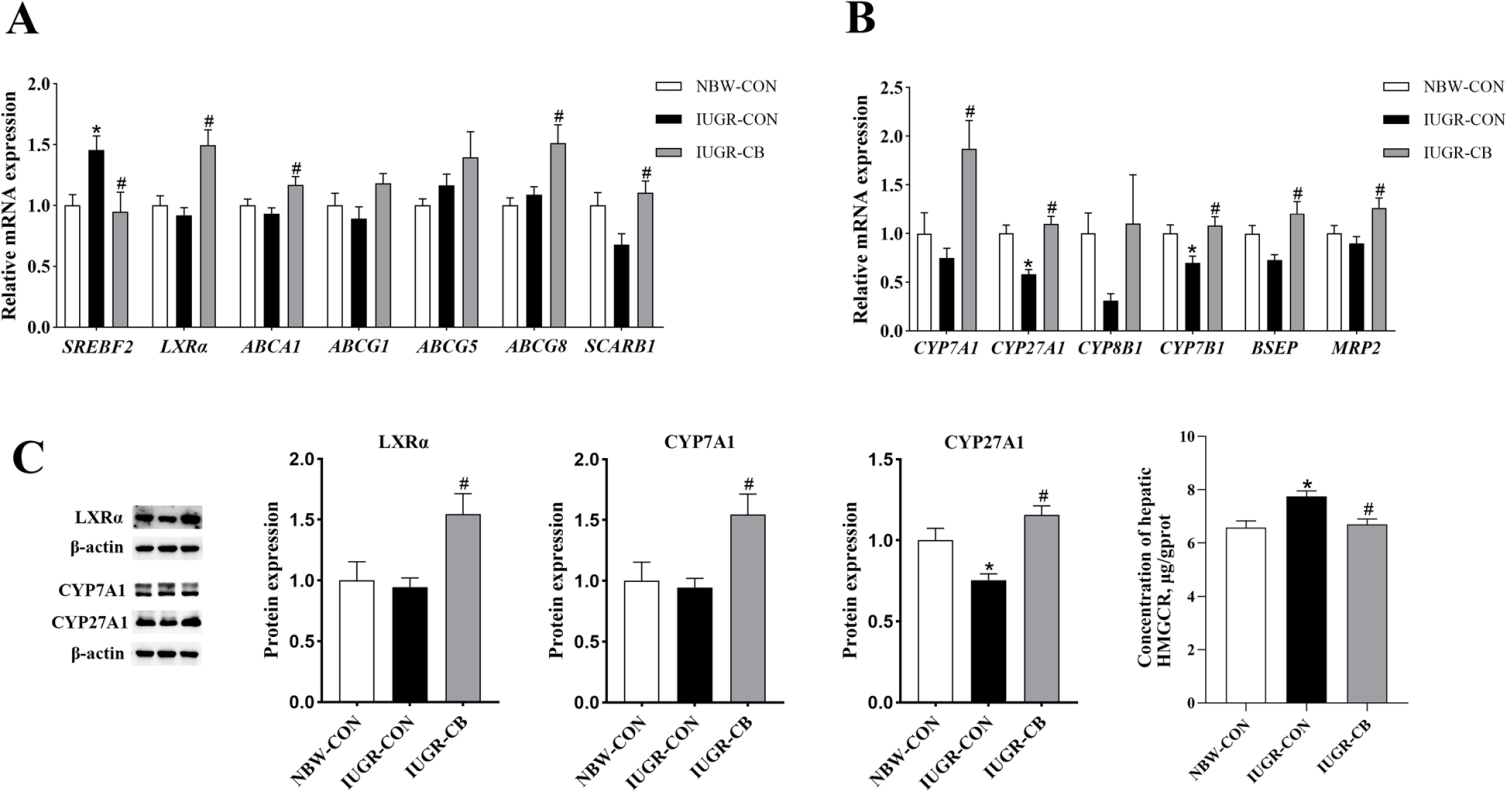
Effect of supplemental *C. butyricum* on cholesterol and BA metabolism of IUGR suckling piglets. **A** The mRNA abundance of genes related to cholesterol synthesis and efflux. **B** The mRNA abundance of genes related to BA synthesis and excretion. **C** The protein levels of LXRα, CYP7A1 and CYP27A1. The column and its bar represented the means value and SE (*n* = 8), respectively. Significant difference is depicted as ^*^P < 0.05 when compared with NBW-CON, ^#^*P* < 0.05 when compared with IUGR-CON group. SREBF2, sterol-regulatory element binding factor 2; LXRα, liver X receptor α; ABCA1, ATP-binding cassette transporter A1; ABCG1, ATP-binding cassette transporter G1; ABCG5, ATP-binding cassette transporter G5; ABCG8, ATP-binding cassette transporter G8; SCARB1, scavenger receptor B class I; CYP7A1, cholesterol 7 α-hydroxylase; CYP27A1, cholesterol 27 α-hydroxylase; CYP7B1, Cytochrome P450 Family 7 Subfamily B Member 1; CYP8B1, Cytochrome P450 Family 8 Subfamily B Member 1; BSEP, bile salt export pump; MRP2, multidrug resistance associated protein 2; NBW-CON, piglets with normal birth weight; IUGR-CON, piglets with intrauterine growth restriction; IUGR-CB, piglets with intrauterine growth restriction supplemented with *Clostridium butyricum*

Compared with the NBW-CON group, the IUGR-CON groups showed significantly higher (*P* < 0.05) *SREBF2* mRNA expression. However, *C. butyricum* supplementation resulted in significantly lower (*P* < 0.05) *SREBF2* expression and, at the same time, higher (*P* < 0.05) expression of genes involved in reverse cholesterol transport and cholesterol efflux, such as *LXRα*, *ABCA1*, *SCARB1* and *ABCG8* (Fig. 3A).

Regarding BA metabolism, the IUGR-CON group exhibited significantly lower (*P* < 0.05) *CYP27A1* mRNA expression compared to the NBW-CON group. However, *C. butyricum* supplementation resulted in higher (*P* < 0.05) expression of genes related to BA synthesis, such as *CYP7A1*, *CYP27A1* and *CYP7B1*, and upregulation (*P* < 0.05) of genes associated with BA excretion, such as *BSEP* and *MRP2* (Fig. 3B).

#### Expression and enzymatic activity of proteins involved in cholesterol metabolism and BA synthesis

Piglets in the IUGR-CON group exhibited lower (*P* < 0.05) HMGCR activity and CYP27A1 expression in the liver compared to piglets in the NBW-CON group. Piglets in the IUGR-CB group exhibited not only elevated (*P* < 0.05) HMGCR activity, but also higher (*P* < 0.05) expression of LXRα, CYP7A1 and CYP27A1 (Fig. 3C).

### Microbial composition of the ileal chyme samples

As is shown in Additional file 2: Table S2, there was no significant difference noted in the α-diversity among the three groups, including in the Sobs index, Chao index, Ace index and Shannon index. However, the IUGR-CB group’s Simpson index was lower (*P* < 0.05) than that of the NBW-CON group. Although no obvious separation was observed between the NBW-CON and IUGR-CON groups, *C. butyricum* treatment did make a difference (*P* < 0.05), as shown in the PCoA (Additional file 4: Fig. S1A) and ANOSIM results (Additional file 4: Fig. S1B).

**Fig. 4:**
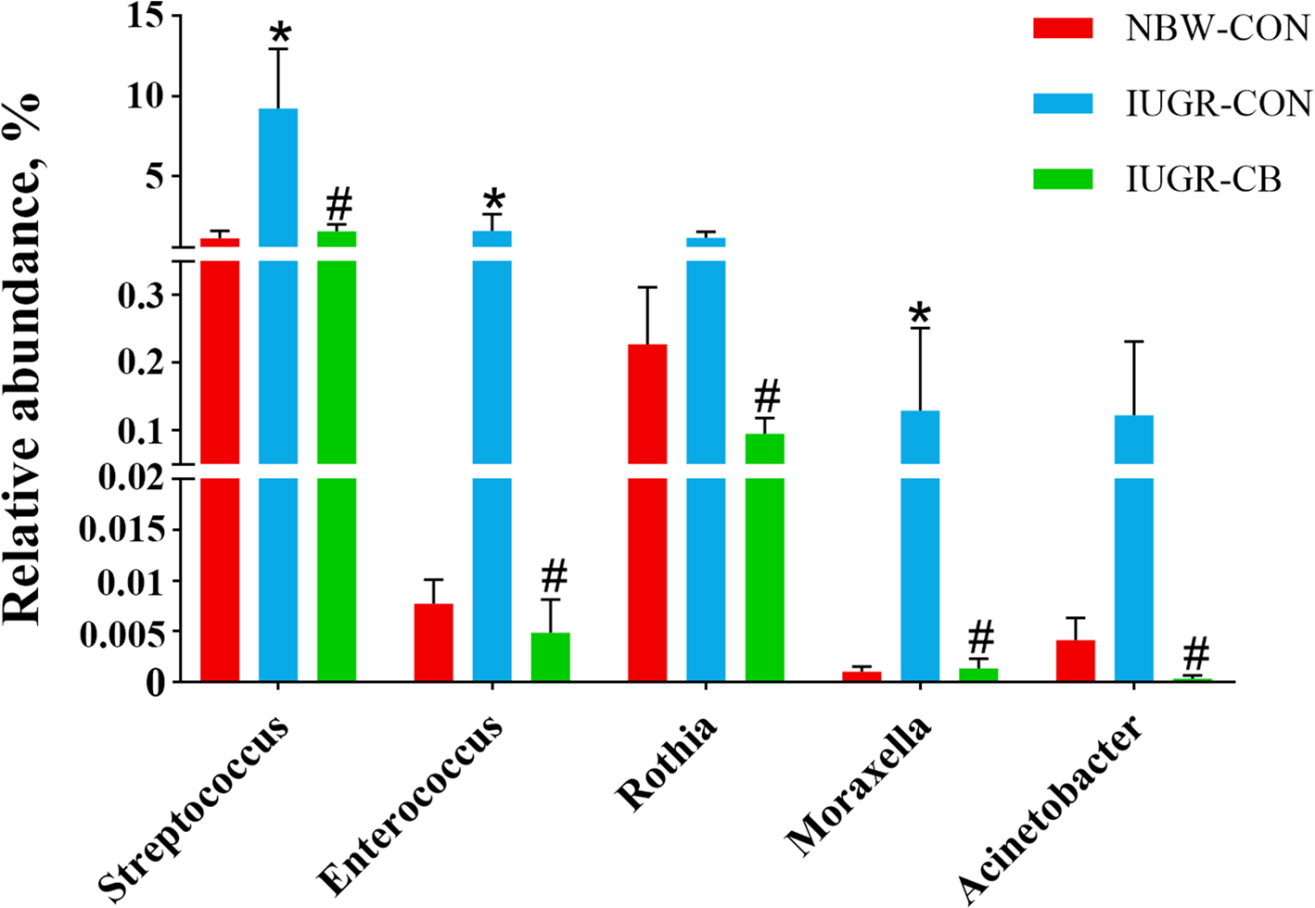
Differences in the relative abundance (> 0.1%) of the piglets’ ileal microbiota at the genus level. The column and its bar represented the means value and SE (*n* = 6), respectively. Significant difference is depicted as ^*^P < 0.05 when compared with NBW-CON, ^#^*P* < 0.05 when compared with IUGR-CON group. *Streptococcus*, *Enterococcus*, *Rothia*, *Moraxella* and *Acinetobacter* are all opportunistic pathogens. Thereinto, *Streptococcus* and *Enterococcus* are BSH-producing microbes. NBW-CON, piglets with normal birth weight; IUGR-CON, piglets with intrauterine growth restriction; IUGR-CB, piglets with intrauterine growth restriction supplemented with *Clostridium butyricum*

At the phylum level (Additional file 4: Fig. S1C), Firmicutes, Proteobacteria and Bacteroidetes predominantly constituted the ileal microbiota of the piglets, and no significant difference was found among the groups. At the genus level (Additional file 4: Fig. S1D), *Lactobacillus*, *Veillonella* and *Actinobacillus* were the dominant genera in the NBW-CON group, while in the IUGR-CON group, *Lactobacillus*, *Streptococcus* and *Escherichia* accounted for the majority of the bacteria. In the IUGR-CB group, the *Lactobacillus*, *Actinobacillus* and *Escherichia* genera dominated. Compared to the NBW-CON group, the IUGR-CON group had a relatively higher abundance of *Streptococcus*, *Enterococcus* and *Moraxella* (*P* < 0.05); however, piglets that received *C. butyricum* had clearly less (*P* < 0.05) *Streptococcus*, *Enterococcus*, *Rothia*, *Moraxella* and *Acinetobacter* compared to piglets in the IUGR-CON group (Fig. 4).

### Intestinal and hepatic BA profiles

**Fig. 5:**
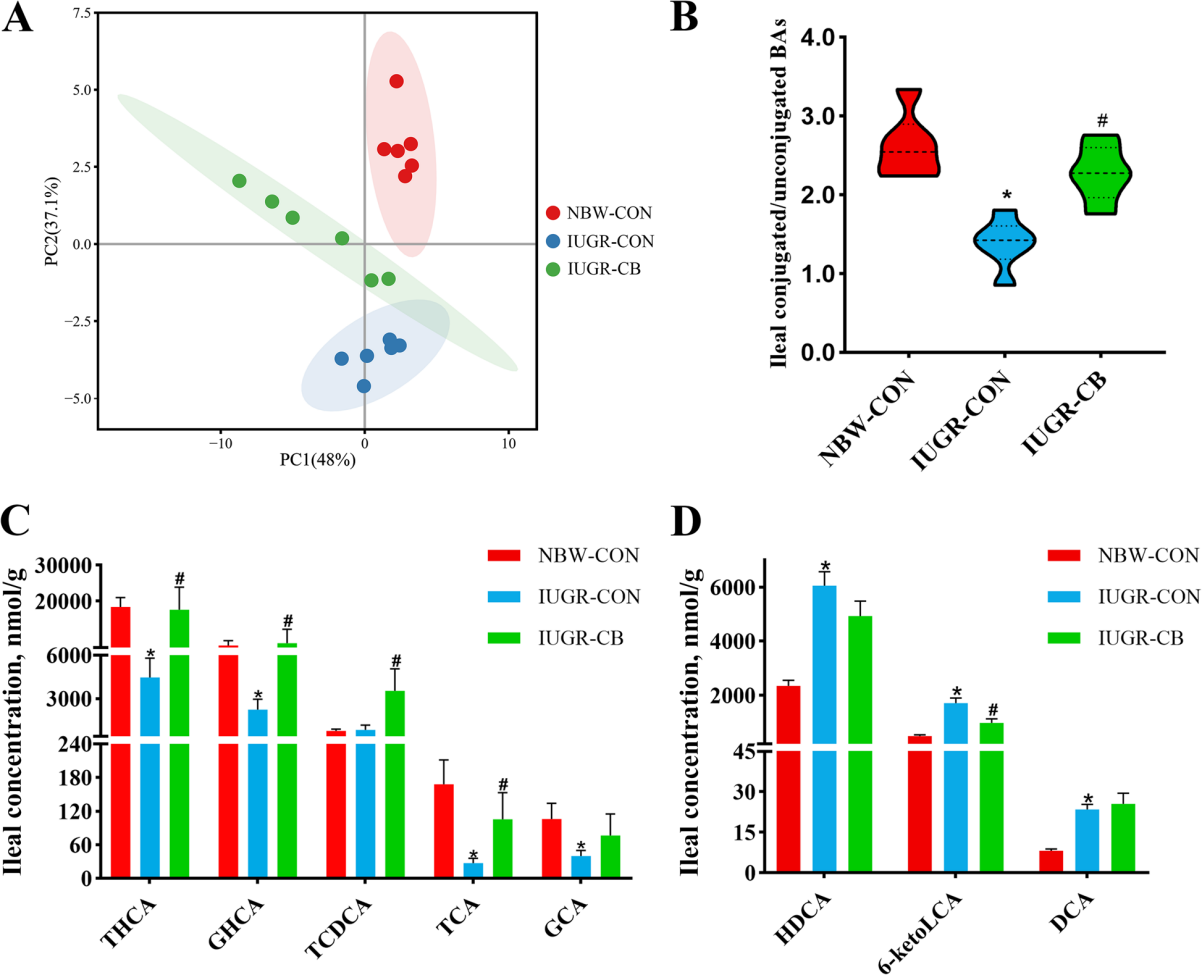
Effect of supplemental *C. butyricum* on ileal BA contents of IUGR suckling piglets. **A** Principal component analysis (PCA) of ileal BAs. **B** The ratio of the content of conjugated BAs to unconjugated BAs. **C** Differential metabolites of conjugated BAs. **D** Differential metabolites of unconjugated BAs. The column and its bar represented the means value and SE (*n* = 6), respectively. Significant difference is depicted as ^*^P < 0.05 when compared with NBW-CON, ^#^*P* < 0.05 when compared with IUGR-CON group. THCA, taurohyocholic acid; GHCA, glycohyocholate; TCDCA, taurochenodeoxycholic acid; TCA, taurocholic acid; GCA, glycocholic acid; HDCA, hyodeoxycholic acid; 6-ketoLCA, 6-ketolithocholic acid; DCA, deoxycholic acid. NBW-CON, piglets with normal birth weight; IUGR-CON, piglets with intrauterine growth restriction; IUGR-CB, piglets with intrauterine growth restriction supplemented with *Clostridium butyricum*

The principal component analysis (PCA) of the ileum samples revealed that there were differences in the ileal BA profiles of the three groups (Fig. 5A). Taurohyocholic acid (THCA), taurohyodeoxycholic acid (THDCA), glycohyocholate (GHCA) and glycohyodeoxycholic acid (GHDCA) accounted for the largest proportion of conjugated BAs in the ileum, while hyocholic acid (HCA), hyodeoxycholic acid (HDCA), and chenodeoxycholic acid (CDCA) represented the major unconjugated BAs in the ileum (Additional file 5: Fig. S2A). Compared to the NBW-CON group piglets, the IUGR-CON group piglets had significantly lower (*P* < 0.05) ratios of conjugated BAs, such as THCA, GHCA, taurocholic acid (TCA) and glycocholic acid (GCA), and higher (*P* < 0.05) levels of unconjugated BAs, such as HDCA, 6-ketolithocholic acid (6-ketoLCA) and deoxycholic acid (DCA) (Fig. 5C, D). As a result, the ratio of conjugated BAs to unconjugated BAs in the ileum of IUGR-CON group piglets decreased significantly (Fig. 5B). Piglets that received *C. butyricum* had dramatically higher (*P* < 0.05) levels of conjugated BAs (THCA, GHCA, taurochenodeoxycholic acid [TCDCA] and TCA), lower (*P* < 0.05) levels of unconjugated BAs (6-ketoLCA), and as a result, a higher ratio between conjugated and unconjugated BAs (Fig. 5B, C, D).

**Fig. 6:**
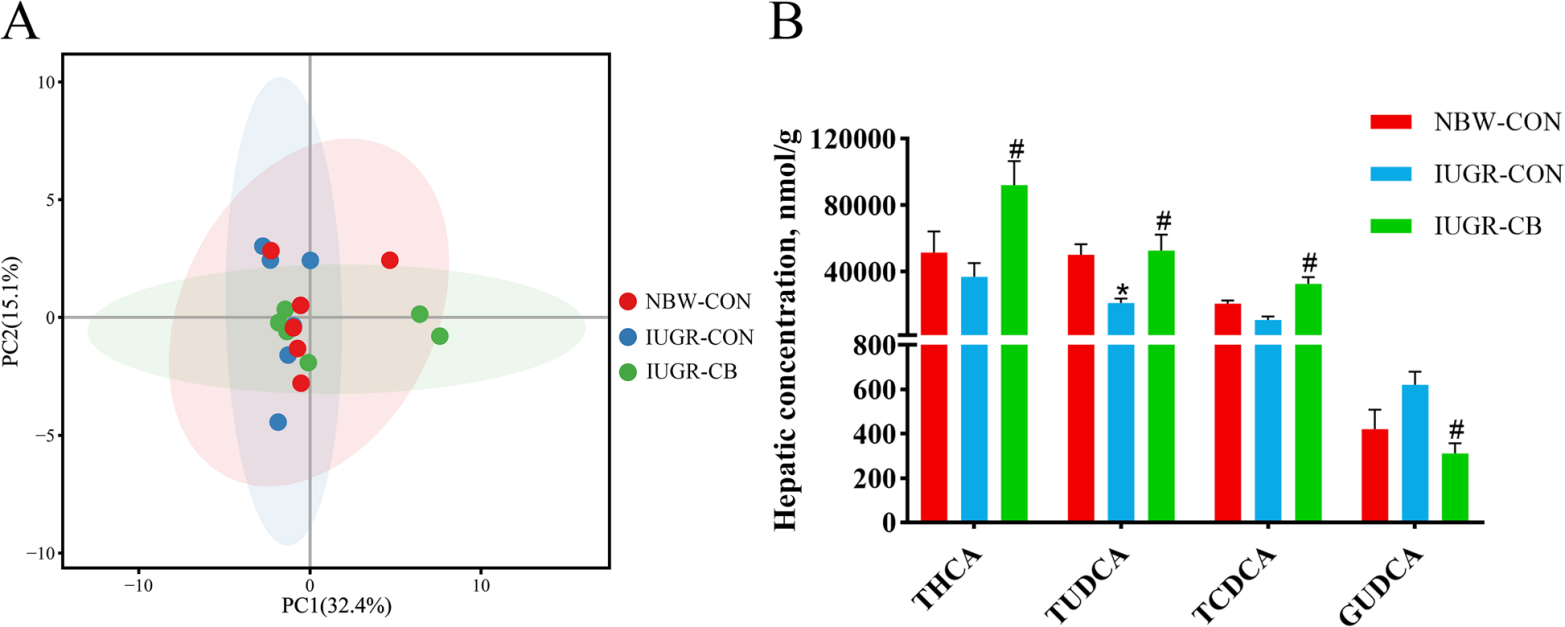
Effect of supplemental *C. butyricum* on hepatic BA contents of IUGR suckling piglets. **A** Principal component analysis (PCA) of ileal BAs. **B** Differential metabolites of hepatic BAs. The column and its bar represented the means value and SE (*n* = 6), respectively. Significant difference is depicted as ^*^P < 0.05 when compared with NBW-CON, ^#^*P* < 0.05 when compared with IUGR-CON group. THCA, Taurohyocholic acid; TUDCA, tauroursodeoxycholic acid; TCDCA, taurochenodeoxycholic acid; GUDCA, glycoursodeoxycholic acid. NBW-CON, piglets with normal birth weight; IUGR-CON, piglets with intrauterine growth restriction; IUGR-CB, piglets with intrauterine growth restriction supplemented with *Clostridium butyricum*

The PCA of the liver samples showed that there was no significant difference among the three groups and that the majority of the hepatic BAs were conjugated BAs (Fig. 6A). GHDCA, GHCA, glycochenodeoxycholic acid (GCDCA), THCA, tauroursodeoxycholic acid (TUDCA), TCDCA and GCA were the primary conjugated BAs found (Additional file 5: Fig. S2B). It was also found that piglets in the IUGR-CON group had significantly lower (*P* < 0.05) TUDCA levels than piglets in the NBW-CON group. In addition, the THCA, TUDCA and TCDCA levels were higher (*P* < 0.05) and the glycoursodeoxycholic acid (GUDCA) level was lower (*P* < 0.05) in the IUGR-CB group compared to the IUGR-CON group (Fig. 6B).

## Discussion

A great deal of evidence shows that the BW of IUGR neonates is significantly lower than that of normal neonates in mammals, and the syndrome has an adverse impact on health over a long period [27]. In the present study, the serum GLU levels (Additional file 3: Table S3) and the FBWs of the IUGR-CON group piglets were significantly lower than those of the NBW-CON group piglets. It is well known that, maintaining serum GLU levels within an appropriate range is necessary for an organism’s metabolic and energy systems to function effectively [28, 29]. Therefore, we could infer that the IUGR-CON group piglets were in a low energy state during the suckling period, and as a result, their growth performance was restricted. Indeed, malnutrition is considered as a leading cause of growth restriction in mammals. When the lacking factor is replenished, spontaneous catch-up growth usually occurs, and brings the individual back to its original growth trajectory [30]. In our study, *C. butyricum* treatment resulted in significantly higher serum GLU levels (Additional file 3: Table S3) compared to the levels in the IUGR-CON piglets, indicating a recovery of the energy supply. As a consequence, the FBWs of the IUGR-CB piglets caught up. Given that the IBWs of IUGR-CB piglets were significantly lower than those of the NBW-CON piglets, the ADGs of IUGR-CB piglets were even higher than those of the NBW-CON piglets. Similarly, other studies have concluded that *C. butyricum* supplementation can effectively improve the growth performance of broilers, weanling pigs and Holstein heifers [31–33].

As a central regulator of lipid homeostasis, the liver is responsible for the de novo synthesis, oxidation and export of FAs; it also controls the biosynthesis and efflux of cholesterol [34, 35]. Maldevelopment of the liver has been observed in IUGR newborns, and this study’s findings of severe damage to the hepatic morphological structure in IUGR infants aligns with those of a previous study [5]. This damage may in turn lead to considerable dysfunction in the lipid metabolism system. Indeed, higher TC levels and lower TBA levels were observed in the livers of the IUGR-CON piglets. BAs are the end products of cholesterol catabolism, and the conversion of cholesterol to BAs accounts for the daily turnover of a major fraction of cholesterol in mammals [36]. These results indicate that IUGR could reduce the transformation of BAs and lead to an excessive accumulation of cholesterol.

Although no significant difference in TG levels was found in either the liver or serum samples, the serum NEFA concentration was notably higher in the IUGR-CON piglets. There is growing evidence that the accumulation of NEFAs is closely associated with a series of health problems, such as obesity, insulin resistance and vascular disease [37–39]. Thus, the elevated NEFA level is likely to have negative effects on the growth and development of these piglets.

In addition, the activity of hepatic HL and the level of serum HDL-C were both lower in the IUGR-CON piglets. HL plays a critical role in the hydrolysis of TGs and promotes the uptake of HDL-C in circulating blood [40, 41]. HDL-C is involved in the reverse cholesterol transport (RCT) pathway, via which excess cholesterol can be transported from the periphery to the liver for clearance [42]. Together, these findings suggest that the ability to clear excess lipids was weakened in the IUGR piglets, which may have increased the risk of developing diseases related to lipid accumulation.

On the contrary, the morphological structure of the liver was normalized in the IUGR-CB piglets. The addition of *C. butyricum* also resulted in less deposition of excess lipids, such as TC and NEFAs, and a simultaneous higher efflux of lipids due to high levels of hepatic TBA, HL and serum HDL-C. Similar effects of *C. butyricum* have been confirmed in HFD mice, indicating that *C. butyricum* intake could effectively improve the HFD-induced accumulation of lipid droplets in hepatocytes and decrease the content of hepatic TC and NEFAs in mice [15]. Intriguingly, we also found that hepatic TG levels in the IUGR-CB group were elevated compared to those in the other two groups. We inferred that this was likely due to a type of feedback mechanism triggered by a high cholesterol level. Because cholesterol esters are less toxic than free cholesterol, the promotion of FA synthesis plays a role in cholesterol homeostasis, with FAs being used as substrates for cholesterol esterification [43]. Hence, as shown above, supplemental *C. butyricum* could effectively regulate the disordered lipid metabolism of IUGR suckling piglets.

To further explore the molecular mechanism of lipid regulation utilized by *C. butyricum*, we detected the levels of genes and proteins associated with FA and cholesterol metabolism. As an important energy substrate, plasma NEFA can provide fuel for mitochondria, the engine of the body, to generate adenosine triphosphate (ATP) [44]. The uptake of circulating FAs by the liver is largely dependent on three major FA transporters located in the hepatocyte plasma membrane: fatty acid transport proteins (FATP), cluster of differentiation 36 (CD36) and caveolins [45, 46]. Following uptake, hydrophobic FAs cannot freely diffuse in the cytosol and must instead be shuttled between different organelles by fatty acid binding proteins (FABP) [45]. In this study, although no significant difference was observed in the expression of *FATP2*, *CD36* and *FABP1* between the NBW-CON and IUGR-CON groups, the expression of *CAV1* mRNA in the IUGR-CON piglets was markedly lower than that in the NBW-CON piglets. However, treatment with *C. butyricum* resulted in not only higher *CAV1* expression, but also higher *CD36* expression. Given that CAV1 contributes to lipid trafficking and lipid droplet formation and that CD36 facilitates long-chain FA (LCFA) transport [46, 47], it could be inferred that the IUGR-CON piglets could not obtain enough materials to generate ATP for their growth and development. However, *C. butyricum* intervention could effectively rectify this issue.

The de novo biogenesis of FAs is mainly controlled by sterol regulatory element-binding protein 1c and its downstream targets ACC and FASN [34]. Thereinto, ACC is the first rate-limiting enzyme that converts acetyl-CoA to malonyl-CoA, and FASN is a key lipogenic enzyme that catalyzes the terminal steps of FAs synthesis [48, 49]. The newly synthesized FAs are then used in TG synthesis, and DGAT1 and DGAT2 catalyze the final step [50]. In the current study, IUGR had no great impact on the de novo synthesis of FAs; however, it reduced the storage of FAs as non-toxic TGs (IUGR-CON group vs. NBW-CON group). In contrast, IUGR piglets treated with *C. butyricum* exhibited increased biosynthesis of FAs and TGs. The reason for this phenomenon could be that IUGR impaired the uptake of FAs, which are an important substrate for TG synthesis. Nevertheless, *C. butyricum* supplementation effectively corrected this and simultaneously promoted FA synthesis. Previous studies have similarly reported that the addition of *C. butyricum* could increase the expression of genes related to FA synthesis in chickens [14, 16].

Following uptake, FAs are utilized by hepatocytes to generate ATP by means of β-oxidation. As the rate-limiting enzyme of FAO, carnitine palmitoyltransferase I (CPT1) catalyzes the conversion of acetyl-CoAs into acyl-carnitines, following which they can cross membranes to enter the mitochondria [51]. PPARα is an FA-activated nuclear receptor that plays a key role in the transcriptional regulation of genes involved in peroxisomal and mitochondrial FAO, such as *ACOX* and *LCAD* [52–54]. In line with the decreased uptake of FAs, the expression of PPARα and its target gene *LCAD* were down-regulated in the IUGR-CON group. Hence, we could infer that the elevated serum NEFA levels probably resulted from the weakened uptake and utilization of FAs in the IUGR-CON piglets. Given the involvement of FAO in energy generation, the growth performance of the IUGR-CON piglets could have been restricted by adverse effects on FAO. However, *C. butyricum* treatment resulted in higher expression of FXR and PPARα and its target genes involved in FAO. As a nuclear receptor, FXR is mainly expressed in the liver and intestine and has a comprehensive effect on lipid metabolism [55]. Similarly, Wang et al. found that *C. butyricum* supplementation could stimulate peroxisomal FA β-oxidation, possibly through the FXR–PPARα–ACOX pathway in hens [14]. Another study with human cells also found that FXR activation induced the expression of PPARα and its downstream genes involved in FAO [56]. It is interesting to note that although mitochondrial FAO was impaired in the IUGR piglets, *C. butyricum* addition improved peroxisomal and mitochondrial FAO both. Substantial evidence supports the notion that mitochondria and peroxisomes exhibit a close functional interplay in the β-oxidation of FAs to maintain lipid homeostasis [57]. Thus, we concluded that the *C. butyricum* treatment greatly improved FAO in the IUGR piglets, and as a result, the serum GLU level was raised and more energy was produced for their growth. To obtain more materials for FAO, the uptake of FAs was accordingly boosted, which likely reduced the serum NEFA content in the IUGR-CB piglets compared to the IUGR-CON piglets and even the NBW-CON piglets.

In cholesterol metabolism, LXRs work together with the sterol regulatory element-binding protein 2 (SREBP2) pathway to maintain cellular and systemic sterol levels [58]. On the one hand, LXRs can facilitate the elimination of excess cholesterol by stimulating biliary cholesterol excretion through the target genes *ABCG5* and *ABCG8* [59]. On the other hand, SREBP2 can boost cholesterol biosynthesis by activating the transcription of the gene that encodes the rate-limiting enzyme HMGCR [60]. The higher expression of *SREBF2* and the activity of HMGCR in the IUGR-CON group compared with that in the NBW-CON group suggested that IUGR promotes cholesterol synthesis, whereas the addition of *C. butyricum* downregulated cholesterol synthesis by decreasing *SREBF2* expression and upregulating its efflux by elevating the expression of LXRα and its downstream target *ABCG8*. LXRα is one isoform of the LXR family that is highly expressed in metabolically active tissues, such as the liver and intestine.

Moreover, LXRα also plays a critical role in promoting RCT, through which excess cholesterol in peripheral tissues can be transferred to HDL and then transported to the liver for BA synthesis and excretion [58]. In this process, downstream genes of LXRα, such as *ABCA1*, *ABCG1* and *SR-BI*, work together to drive the assembly of HDL to initiate RCT [61–63]. Then, the excess cholesterol transported by HDL-C is used for BA synthesis, which is critical for maintaining cholesterol homeostasis and preventing the accumulation of cholesterol in the liver [36].

BAs are synthesized by multi-step reactions catalyzed in hepatocytes via two distinct routes: the “classical” (neutral) pathway and the “alternative” (acidic) pathway. The classic pathway is initiated by 7α-hydroxylation of cholesterol catalyzed by the rate-limiting enzyme CYP7A1, followed by further transformations of the steroid nucleus and oxidative cleavage of the side chain involving CYP8B1 [64]. The alternative pathway is initiated by sterol 27-hydroxylase (CYP27A1). This reaction is followed by oxysterol 7α-hydroxylation, which is primarily mediated by CYP7B1 [65]. Finally, the synthesized BAs are secreted through the bile canalicular membrane by two ABC transporters (BSEP and MRP2) into the canalicular lumen [66]. Consistent with the variation in hepatic TBA levels, the expression of CYP27A1 and *CYP7B1* was lower in the IUGR-CON group compared to that in the NBW-CON group. In the IUGR-CB group, expression of CYP7A1 and CYP27A1 and its downstream gene *CYP7B1* was markedly higher, and *BSEP* and *MRP2* expression were both increased accordingly. These results revealed that the reduced BA levels in the IUGR-CON group were likely the result of an impaired alternative pathway, and that *C. butyricum* treatment could effectively restore BA content to normal levels by promoting the classic and alternative pathways. A study that focused on oxysterol 7α-hydroxylase, an important enzyme in the alternative pathway, confirmed the quantitative importance of the alternative pathway in early life in humans [67], suggesting that the alternative pathway might be the major route of BA synthesis in infants. Given the specific period of suckling, malfunction of the alternative pathway would consequently lead to considerable disruption of cholesterol metabolism. Moreover, previous studies have similarly indicated that increases in CYP27A1 activity could downregulate cholesterol synthesis through the SREBP pathway as well as enhance the efflux and elimination of cholesterol via LXR [68].

There is a plethora of evidence confirming that the composition of the gut microbiota can have profound effects on the host [69, 70]. In our study, no significant difference was found in the microbial α-diversity among the three groups except for a decreased Simpson index in the IUGR-CB group compared to the NBW-CON group. Hence, it could be inferred that the addition of *C. butyricum* affected the homogeneity of the gut microbiota by modulating its composition. To further investigate the connection between the change in the gut microbiota and the effect of lipid regulation in the IUGR-CB group, we analyzed the differences in the bacteria among the groups at the phylum and genus levels. Although no significant difference was found at the phylum level, piglets in the IUGR-CON group had significantly more opportunistic pathogens, such as *Streptococcus*, *Enterococcus* and *Moraxella*, which increases the risk of developing an inflammatory response and associated impairment of normal liver function [71–73]. Intriguingly, *C. butyricum* treatment resulted in not only lower relative abundance of the bacteria mentioned above but also lower relative abundance of *Rothia* and *Acinetobacter*, which are also considered opportunistic pathogens that could have negative effects on the host’s health [74, 75]. Hence, the modulation of the gut microbiota caused by *C. butyricum* might have effectively protected the IUGR piglets against pathogen invasion, and this is supported by the observed recovery of congestion in the liver portal vein and sinusoids.

Of note, among the changed microbes, *Streptococcus* and *Enterococcus* are BSH-producing microbes; BSH catalyzes the deconjugation of glycine- or taurine-conjugated BAs to form unconjugated BAs [76, 77]. As a result, the ileal BA profiles of the IUGR-CON piglets were altered compared with those of the NBW-CON piglets, and the profiles were characterized by lower levels of conjugated BAs and higher levels of unconjugated BAs. It is well known that BAs can function as endogenous signaling molecules by binding to BA receptors, such as FXR and LXRα, to regulate BA homeostasis in enterohepatic circulation and to modulate cholesterol and TG metabolism [36, 78]. In the current study, *C. butyricum* treatment was found to elevate the proportion of conjugated BAs in the ileum, and the THCA, TCDCA and TCA levels were dramatically increased. THCA is a known LXRα agonist, and TCA and TCDCA are known FXR agonists [23, 79]. Therefore, these signaling molecules might be transported to the liver via enterohepatic circulation and then play an important role in regulating lipid metabolism. To confirm this, we also analyzed the BA profile in the liver. In line with the results derived from the ileum, *C. butyricum* supplementation increased the levels of the LXRα agonist THCA and the FXR agonist TCDCA. Meanwhile, GUDCA, an FXR antagonist [80], was also decreased in the IUGR-CB group compared with the IUGR-CON group. Hence, FXR and LXRα may have been simultaneously activated to affect lipid metabolism in the liver.

**Fig. 7:**
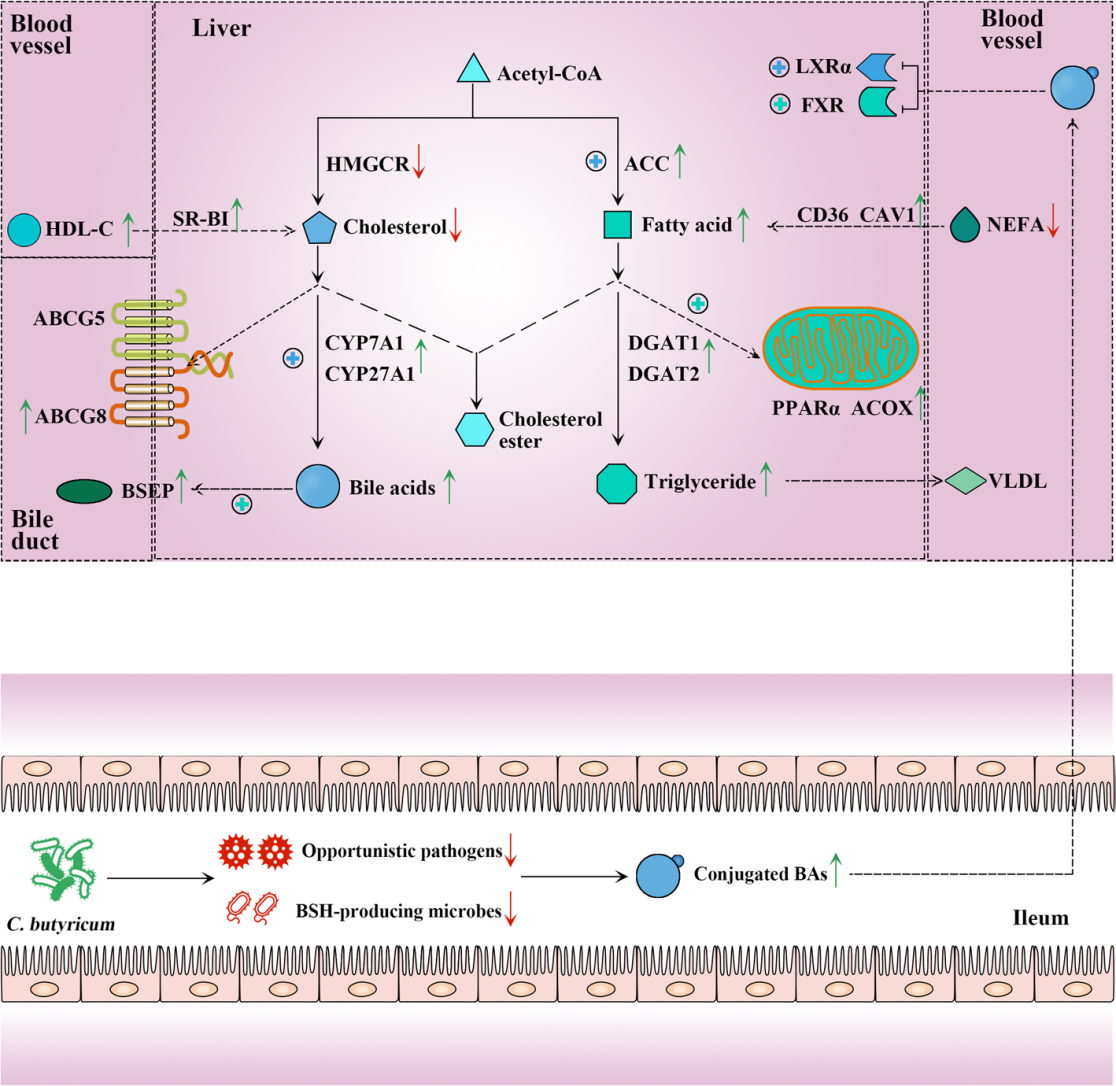
Supplemental *C. butyricum* could effectively improve lipid disorders of IUGR suckling piglets. *C. butyricum* treatment modulated the gut microbiota to reduce the relative abundance of opportunistic pathogens in IUGR piglets. Therein, *Streptococcus* and *Enterococcus* are BSH-producing microbes, so that some conjugated BAs function as signaling molecules were increased in the ileum. Via enterohepatic circulation, these signaling molecules could be transported to the liver to activate LXRα and FXR. The activation of LXRα could promote the synthesis of FAs and the transformation of cholesterol to BAs, and the activation of FXR could increase the β-oxidation of FAs and the excretion of BAs.

Although FXR activation can repress BA synthesis, it relies on the effect of small heterodimer partner (SHP). The importance of SHP in the feedback regulation of BA synthesis was demonstrated in SHP^−/−^ mice, in which the repression of CYP7A1 was dismissed and the size of the BA pool was enlarged [81]. In our study, despite the activation of FXR, no significant difference in *SHP* expression was observed in the IUGR-CB group compared to the IUGR-CON group. As a result, BA synthesis may not have been repressed. Conversely, the synthesis of BAs may have been upregulated by the activation of LXRα. As a type of hydrophilic BA used to treat hepatobiliary disorders, TUDCA can penetrate into the cell membrane and help transfer cholesterol from the cell membrane to HDL [82, 83]. A previous study showed that TUDCA treatment could effectively decrease serum and hepatic TC levels and increase the mRNA expression of *CYP27A1* in a model of cholesterol gallstones [84]. Similarly, in our study, and consistent with the lower hepatic TUDCA levels in the IUGR piglets, the serum HDL-C level was lower and the hepatic TC level was higher in the IUGR-CON group than in the NBW-CON group. However, *C. butyricum* supplementation resulted in significantly higher hepatic TUDCA levels and, at the same time, promoted cholesterol efflux by increasing the serum HDL-C level to drive the transport of cholesterol and upregulating the expression of CYP27A1 to accelerate BA synthesis (Fig. 7).

## Conclusion

Based on the findings of this study, we concluded that providing *C. butyricum* to IUGR suckling piglets might result in modulation of the gut microbiota; specifically, a reduction in the relative abundance of BSH-producing microbes, such as *Streptococcus* and *Enterococcus*. Thus, the levels of some conjugated BAs that function as signaling molecules were increased in the ileum. Via enterohepatic circulation, these signaling molecules could be transported to the liver and further regulated hepatic lipid metabolism by activating LXRα and FXR. As a result, the lipid metabolism was normalized and the growth performance was improved in the IUGR suckling piglets.

## Supplementary Information

**Additional file 1: Table S1.** Sequences for real-time PCR primers.

**Additional file 2: Table S2.** Effect of supplemental *C. butyricum* on alpha diversity of ileal microbiota in IUGR suckling piglets.

**Additional file 3: Table S3.** Effect of supplemental *C. butyricum* on serum GLU of IUGR suckling piglets.

**Additional file 4: Fig. S1.** Effect of supplemental *C. butyricum* on the microbial structure of the ileum in IUGR suckling piglets.

**Additional file 5: Fig. S2.** Effect of supplemental *C. butyricum* on BAs composition of the ileum and liver.

## Data Availability

The datasets generated and/or analyzed during the present study are only available from the corresponding author on reasonable request.

## Abbreviations

ABCA1: ATP-binding cassette transporter A1
ABCG1: ATP-binding cassette transporter G1
ABCG5: ATP-binding cassette transporter G5
ABCG8: ATP-binding cassette transporter G8
ACC: Acetyl-CoA carboxylase
ACOX: Acyl-CoA oxidase
ADG: Average daily gain
BA: Bile acids
BSEP: Bile salt export pump
BSH: Bile salt hydrolase
CAV1: Caveolin 1
*C. butyricum*: *Clostridium butyricum*
CD36: Cluster of differentiation 36
CPT1: Carnitine palmitoyltransferase 1
CYP27A1: Cholesterol 27 α-hydroxylase
CYP7A1: Cholesterol 7alpha-hydroxylase
CYP7B1: Cytochrome P450 Family 7 Subfamily B Member 1
CYP8B1: Cytochrome P450 Family 8 Subfamily B Member 1
DGAT1: Diacylglycerol acyltransferase 1
DGAT2: Diacylglycerol acyltransferase 2
FA: Fatty acid
FABP: Fatty acid binding protein
FASN: Fatty acid synthase
FBW: Final body weight
FXR: farnesoid X receptor
GHCA: Glycohyocholate
GUDCA: Glycoursodeoxycholic acid
HDL-C: High-density lipoprotein cholesterol
HL: Hepatic lipase
HMGCR: 3-hydroxy-3-methylglutaryl-CoA reductase
IUGR: Intrauterine growth retardation
LCAD: Long-chain acyl-CoA dehydrogenase
LXRα: Liver X receptor alpha
MRP2: Multidrug resistance associated protein 2
NBW: Normal birth weight
NEFA: Nonesterified free fatty acids
PPARα: peroxisome proliferator activated receptor alpha
SREBF2: Sterol-regulatory element binding factor 2
TC: Total cholesterol
TCA: Taurocholic acid
TCDCA: Taurochenodeoxycholic acid
TG: Triglyceride
THCA: Taurohyocholic acid
TUDCA: Tauroursodeoxycholic acid
