# Supplementary material for: Supplemental Clostridium butyricum modulates lipid metabolism by reshaping the gut microbiota composition and bile acid profile in IUGR suckling piglets
Source: J Anim Sci Biotechnol. 2023 Mar 13;14:36. doi: 10.1186/s40104-023-00828-1 (PMC10009951; doi:10.1186/s40104-023-00828-1)
Supplement: Supplementary file 1 — Additional file 1: Table S1. Sequences for real-time PCR primers. [file 40104_2023_828_MOESM1_ESM.docx]

**Supplementary Table. 1** Sequences for real-time PCR primers

| Genes | Accession No. | Sequences |
| --- | --- | --- |
| *β-actin* | XM_003124280.5 | F: GCCAGAAGGACTCCTACGTG  R: CATGTCGTCCCAGTTGGTGA |
| *FATP* | JX092264 | F: GGACGAGACGCTCACCTATG  R: CCAGCCAGAGCCACACATAA |
| *CD36* | NM_001044622 | F: CTGGCCGTGTTTGGAGGTAT  R: TCCGTGCCTGTTTTAACCCA |
| *CAV1* | NM_001348935 | F: CTCCTTCCTGCACATCTGGG |
|  |  | R: AAAGAGCGGGTCACAGAAGG |
| *FABP* | NM_001004046 | F: GCCAGGAAAACTTTGAGGCC  R: TGCTTCCCATTCTGCACGAT |
| *SREBP1c* | NM_214157 | F: TGCACACCCAGGTTCAAAGT  R: AGTCCGCCTTGATGAAGTGG |
| *ACC* | NM_001114269 | F: GTGCTGTTGGACAACGCATT  R: GGGGTGGGGAATCACAGAAG |
| *FASN* | NM_001099930 | F: CGTTGGGTCGACTCACTGAA  R: GAGACAGTTCACCATGCCCA |
| *DGAT1* | NM_214051 | F: TACTACTTCCTCCTGGCCCC  R: TGCAGCTGGATGAGGAACAG |
| *DGAT2* | NM_001160080 | F: GGGTCCTGTCTTTCCTCGTG  R: CGCCAGCCAAGTGAAGTAGA |
| *FXR* | KF597010 | F: TGACAAAGACGACCCGACTG  R: AAACCTTTGCACCCCTCACA |
| *CPT1* | NM_001129805 | F: CAAGATAGCGGCCGAAAAGC  R: GGCGAGGTATTTGGACACCA |
| *PPARα* | NM_001044526 | F: GCAATAACCCGCCTTTCGTC  R: CTCCTTGTTCTGGATGCCGT |
| *LCAD* | D89478 | F: CGCTGCTTCCATGGCAAAAT  R: AAGCTCTGGCAATGGGGTAC |
| *ACOX* | NM_001101028 | F: CCCGCCTGGAACTTCAAGAT  R: GCAGGGTCATAAGTGGCTGT |
| *SREBF2* | XM_021091444 | F: AAGAAGGAGAAAGGCGGACG  R: GGCATCTGTTCCCATGACCA |
| *LXRα* | AB254405 | F: CCCAGCAGCAGTGTAACAGA  R: GATGGCCAGCTCAGTGAAGT |
| *ABCA1* | NM_001317080 | F: CGTCTCCGCTATCTCCAACC  R: CACAAAGGCTCCCTCTCTGG |
| *ABCG1* | XM_021071021 | F: ATCCTCAGAGAGCTGGACGT  R: GGCCCGGATTTTGTACCTCA |
| *ABCG5* | XM_021087571 | F: TGAACCACGTTGCAGATCGA  R: CAGTCTAGGCCTGTGGTTGG |
| *ABCG8* | XM_021087569 | F: CTGGGCCTTCAGAACCTGAG  R: CGACCGGTGATCACGTCTAG |
| *SCARB1* | NM_213967 | F: CTGACCCAGTTCTGGCAGAG  R: GCAGTTCATAGGGATCCCGG |
| *CYP7A1* | KP687249 | F: AGCATTGACCCCAGTGATGG  R: GGGGTCTCAGGACAAGTTGG |
| *CYP27A1* | NM_001243304 | F: CCTTCGTCAGATCTGTCGGG  R: ATCCAGGTATCGCCTCCAGT |
| *CYP8B1* | NM_214426 | F: TGCACATGGACCCTGACATC  R: GTGGATCTTCTTGCCTGCCT |
| *CYP7B1* | XM_021089296 | F: TTGGACAGCCTGGTCTACCT  R: ACAAAGTCTCCCTTCCGCAG |
| *BSEP* | XM_003133457 | F: TATTGCTCGGGCCATCGTAC  R: CCGACCTTCTCTGGCTTTGT |
| *MRP2* | XM_021073710 | F: TCCTACGAGGTGACAGAGGG  R: GTCTCTAGATCCACCGCAGC |
