# Supplementary material for: Supplemental Clostridium butyricum modulates lipid metabolism by reshaping the gut microbiota composition and bile acid profile in IUGR suckling piglets
Source: J Anim Sci Biotechnol. 2023 Mar 13;14:36. doi: 10.1186/s40104-023-00828-1 (PMC10009951; doi:10.1186/s40104-023-00828-1)
Supplement: Supplementary file 2 — Additional file 2: Table S2. Effect of supplemental C. butyricum on alpha diversity of ileal microbiota in IUGR suckling piglets. [file 40104_2023_828_MOESM2_ESM.docx]

**Supplementary Table. 2** Effect of supplemental *C. butyricum* on alpha diversity of ileal microbiota in IUGR suckling piglets

| Items | NBW-CON | IUGR-CON | IUGR-CB | *P* values | | |
| --- | --- | --- | --- | --- | --- | --- |
|  |  |  |  | 1 | 2 | 3 |
| Sobs index | 221.50 ± 56.30 | 249.33 ± 51.93 | 176.50 ± 24.01 | 0.748 | 0.872 | 0.262 |
| Chao index | 296.74 ± 51.58 | 350.94 ± 59.23 | 271.49 ± 35.93 | 0.394 | 0.699 | 0.240 |
| Ace index | 350.19 ± 49.61 | 405.25 ± 72.38 | 396.33 ± 43.25 | 0.589 | 0.699 | 0.818 |
| Shannon index | 1.71 ± 0.33 | 2.36 ± 0.32 | 2.23 ± 0.15 | 0.180 | 0.132 | 0.818 |
| Simpson index | 0.41 ± 0.08 | 0.21 ± 0.06 | 0.19 ± 0.05* | 0.132 | 0.026 | 0.937 |

^1^Sobs, observed species.

^2^NBW-CON, piglets with normal birth weight; IUGR-CON, piglets with intrauterine growth restriction; IUGR-CB, piglets with intrauterine growth restriction supplemented with *Clostridium butyricum*.

All data are presented as mean ± SE (*n* = 6). Significant difference is depicted as * *P* < 0.05 when compared with NBW-CON, **^#^** *P* < 0.05 when compared with IUGR-CON group. Contrast: (1) NBW-CON versus IUGR-CON; (2) NBW-CON versus IUGR-CB; (3) IUGR-CON versus IUGR-CB
