# Supplementary material for: Supplemental Clostridium butyricum modulates lipid metabolism by reshaping the gut microbiota composition and bile acid profile in IUGR suckling piglets
Source: J Anim Sci Biotechnol. 2023 Mar 13;14:36. doi: 10.1186/s40104-023-00828-1 (PMC10009951; doi:10.1186/s40104-023-00828-1)
Supplement: Supplementary file 3 — Additional file 3: Table S3. Effect of supplemental C. butyricum on serum GLU of IUGR suckling piglets. [file 40104_2023_828_MOESM3_ESM.docx]

| Items | NBW-CON | IUGR-CON | IUGR-CB | *P* values | | |
| --- | --- | --- | --- | --- | --- | --- |
|  |  |  |  | 1 | 2 | 3 |
| GLU,  mmol/L | 5.78 ± 0.68 | 3.38 ± 0.47* | 7.11 ± 0.56**^#^** | 0.022 | 0.253 | <0.001 |

**Supplementary Table. 3** Effect of supplemental *C. butyricum* on serum GLU of IUGR suckling piglets

^1^GLU, glucose.

^2^NBW-CON, piglets with normal birth weight; IUGR-CON, piglets with intrauterine growth restriction; IUGR-CB, piglets with intrauterine growth restriction supplemented with *Clostridium butyricum*.

All data are presented as mean ± SE (*n* = 8). Significant difference is depicted as * *P* < 0.05 when compared with NBW-CON group, **^#^** *P* < 0.05 when compared with IUGR-CON group. Contrast: (1) NBW-CON versus IUGR-CON; (2) NBW-CON versus IUGR-CB; (3) IUGR-CON versus IUGR-CB.
