# Supplementary material for: Supplemental Clostridium butyricum modulates lipid metabolism by reshaping the gut microbiota composition and bile acid profile in IUGR suckling piglets
Source: J Anim Sci Biotechnol. 2023 Mar 13;14:36. doi: 10.1186/s40104-023-00828-1 (PMC10009951; doi:10.1186/s40104-023-00828-1)
Supplement: Supplementary file 4 — Additional file 4: Fig. S1. Effect of supplemental C. butyricum on the microbial structure of the ileum in IUGR suckling piglets. [file 40104_2023_828_MOESM4_ESM.docx]

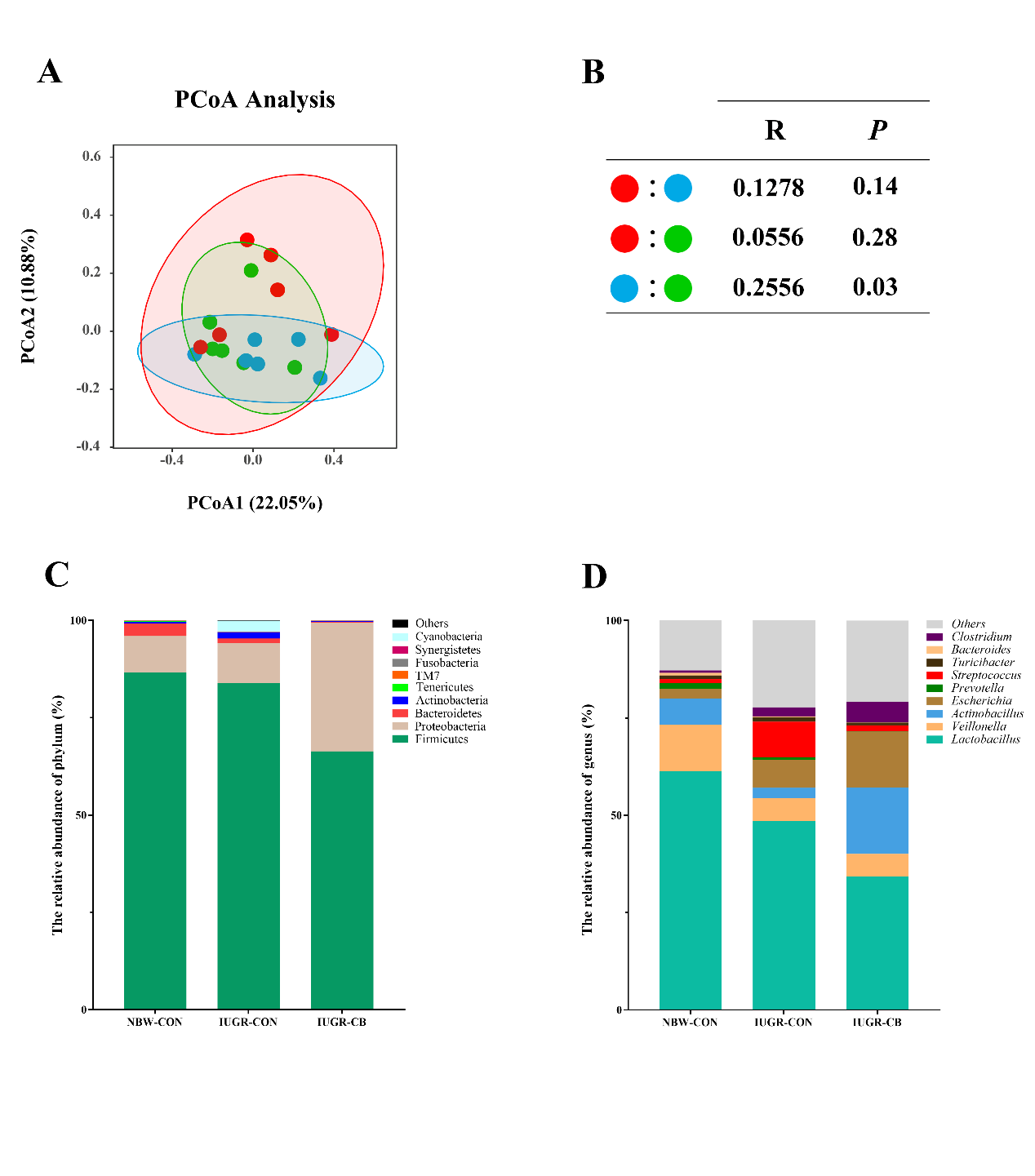


Supplementary Fig. 1 Effect of supplemental *C. butyricum* on the microbial structure of the ileum in IUGR suckling piglets. **a** Principal coordinate analysis (PCoA) of all samples. **b** Analysis of similarities (ANOSIM) of the three treatments. **c** The gut microbiota composition at the phylum level. **d** The gut microbiota composition at the genus level. Data are shown as means, *n* = 6. NBW-CON, piglets with normal birth weight; IUGR-CON, piglets with intrauterine growth restriction; IUGR-CB, piglets with intrauterine growth restriction supplemented with *Clostridium butyricum*.
