# Supplementary material for: Supplemental Clostridium butyricum modulates lipid metabolism by reshaping the gut microbiota composition and bile acid profile in IUGR suckling piglets
Source: J Anim Sci Biotechnol. 2023 Mar 13;14:36. doi: 10.1186/s40104-023-00828-1 (PMC10009951; doi:10.1186/s40104-023-00828-1)
Supplement: Supplementary file 5 — Additional file 5: Fig. S2. Effect of supplemental C. butyricum on BAs composition of the ileum and liver. [file 40104_2023_828_MOESM5_ESM.docx]

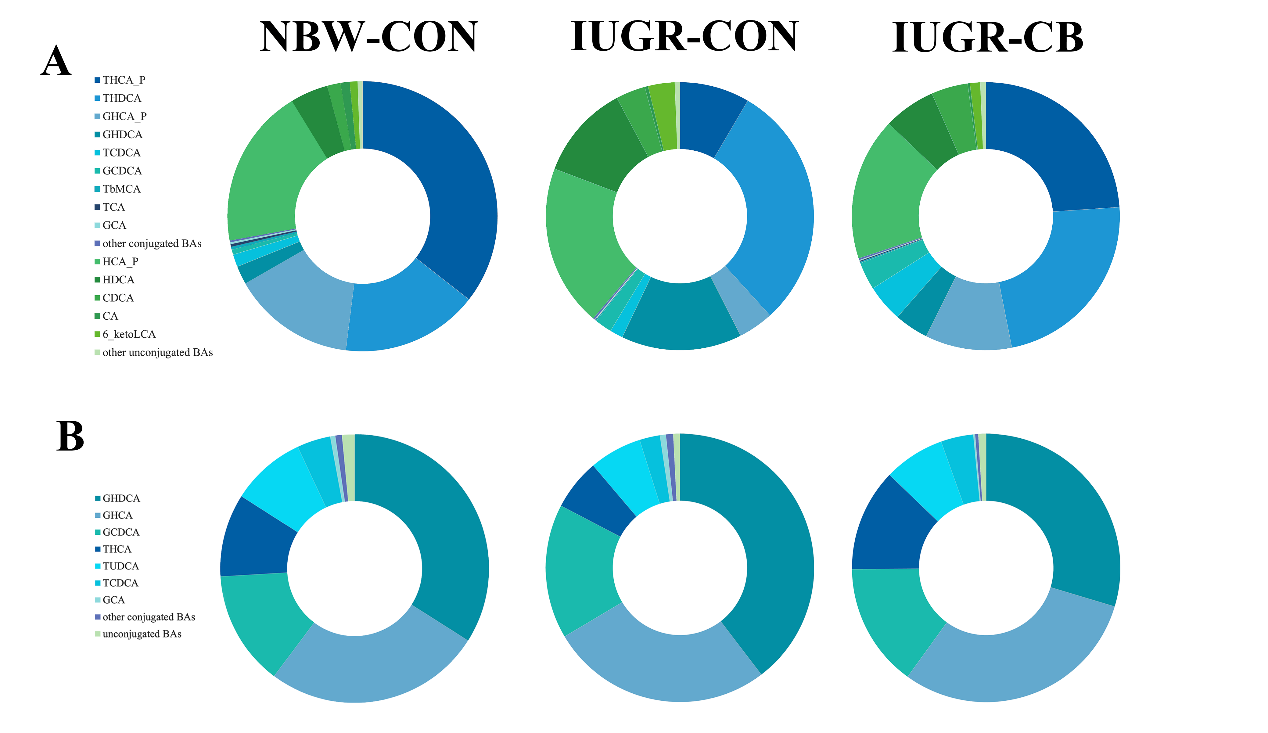


Supplementary Fig. 2 Effect of supplemental *C. butyricum* on BAs composition of ileum (A) and liver (B). Data are shown as means, *n* = 6. THCA, taurohyocholic acid; THDCA, taurohyodeoxycholic acid; GHCA, glycohyocholate; GHDCA, glycohyodeoxycholic acid; TCDCA, taurochenodeoxycholic acid; GCDCA, glycochenodeoxycholic acid; TβMCA, tauro β-muricholic acid; TCA, taurocholic acid; GCA, glycocholic acid; HCA, hyocholic acid; HDCA, hyodeoxycholic acid; CDCA, chenodeoxycholic acid; CA, cholic acid; 6-ketoLCA, 6-ketolithocholic acid; TUDCA, tauroursodeoxycholic acid. NBW-CON, piglets with normal birth weight; IUGR-CON, piglets with intrauterine growth restriction; IUGR-CB, piglets with intrauterine growth restriction supplemented with *Clostridium butyricum*.
